# Preterm Birth in Women With HIV: The Role of the Placenta

**DOI:** 10.3389/fgwh.2022.820759

**Published:** 2022-03-15

**Authors:** Nadia M. Ikumi, Mushi Matjila

**Affiliations:** Department of Obstetrics and Gynaecology, University of Cape Town, Cape Town, South Africa

**Keywords:** HIV - human immunodeficiency virus, placenta, decidua, dNK cells, macrophage, T cells, Treg, preterm (birth)

## Abstract

Maternal HIV infection is associated with an increased risk of preterm birth (PTB). However, the mechanisms underlying this increased risk in women with HIV remain poorly understood. In this regard, it is well-established that labor is an inflammatory process and premature activation of the pro-inflammatory signals (associated with labor) can result in preterm labor which can subsequently lead to PTB. HIV infection is known to cause severe immune dysregulation within its host characterized by altered immune profiles, chronic inflammation and eventually, the progressive failure of the immune system. The human placenta comprises different immune cell subsets, some of which play an important role during pregnancy including participating in the inflammatory processes that accompany labor. It is therefore plausible that HIV/antiretroviral therapy (ART)-associated immune dysregulation within the placental microenvironment may underlie the increased risk of PTB reported in women with HIV. Here, we review evidence from studies that point toward the placental origin of spontaneous PTB and discuss possible ways maternal HIV infection and/or ART could increase this risk. We focus on key cellular players in the maternal decidua including natural killer cells, CD4+ T cells including CD4+ regulatory T cells, CD8+ T cells as well as macrophages.

## Introduction

Preterm birth (PTB) is defined by the World Health Organisation (WHO) as delivery before 37 completed weeks of gestation (1). Extreme PTB is defined as delivery before 28 weeks of gestation, very PTB as delivery between 28 and 32 weeks' gestation and moderate or late PTB as delivery between 32 and 36 weeks' gestation (1). New global estimates on PTB, published in 2018, indicate there were an estimated 14.8 million global live PTBs in 2014 (2). The highest rates of PTB were in low- and middle-income countries (LMICs) particularly in Asia, and sub-Saharan Africa (SSA) which accounted for 12 million PTBs (2). PTB is a leading cause of mortality as it accounts for ~8,500,000 deaths in the neonatal period (first 28 days of life) and an estimated 106,000 deaths in children aged 1–59 months, globally (3). The rates of morbidity and mortality in preterm neonates increase with the decrease in gestational age (4). There are also significantly increased associated economic costs as well as psychosocial and physical burden when neonates are born at earlier gestations (4). The hardest hit in this regard are LMICs, particularly where births occur in facilities lacking the infrastructural resources and expertise to provide quality obstetric and neonatal care.

**Figure 1:**
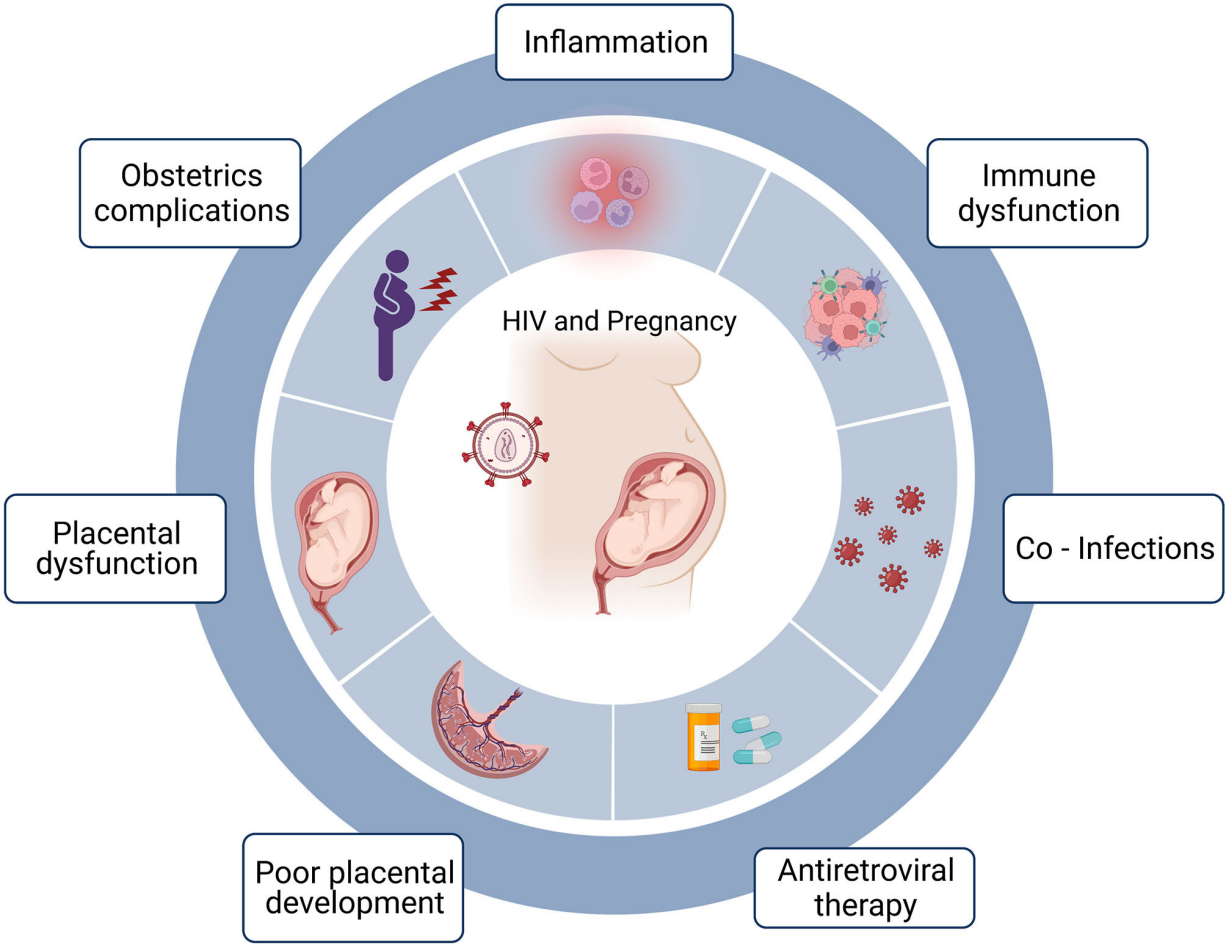
HIV, pregnancy and preterm birth. Maternal HIV infection is associated with preterm birth and the factors that may be linked to preterm birth include acute and chronic inflammation often associated with HIV infection. Immune dysfunction including skewed T cell differentiation, perturbed effector function and altered cellular homeostasis. Co-infections including opportunistic infections due to immunodeficiencies. Certain antiretroviral treatment regimens have been linked to preterm birth and timing of treatment initiation can impact birth outcomes. Poor placental development possibly linked to defective deep placentation. Placental dysfunction including poor perfusion and placental insufficiency and obstetric complications. Created in Biorender.com.

Maternal HIV infection is associated with an increased risk of PTB (5, 6). UNAIDS estimates indicate that in 2020, there were 15.5 million women (aged 15–49 years) with HIV globally (7). HIV infection is known to cause severe immune dysregulation within its host characterized by altered CD4:CD8 T cell ratios, chronic inflammation and progressively, the eventual failure of the immune system (8–10). The human placenta comprises different immune cell subsets, some of which play an important role during pregnancy including participating in the inflammatory processes that accompany labor (11). These include decidual natural killer cells, CD4+ T, CD8+ T cells, regulatory T cells as well as macrophages (11). It is therefore plausible that HIV/antiretroviral therapy (ART)-associated immune dysregulation within the placental microenvironment may underlie the increased risk of PTB reported in women with HIV (Figure 1). Indeed, several studies have demonstrated the adverse effects HIV/ART can have on the placenta. Placentae from women with HIV have been shown to have increased features of placental insufficiency including maternal vascular malperfusion in addition to acute and chronic inflammation (12). These patterns of injury have further been associated with adverse birth outcomes such as PTB and available evidence links poor placental function to compromised fetal development (13, 14). Women with HIV manifest an altered placental T cell profile where the decidua appears to mirror the systemic circulation during HIV infection characterized by a dysregulated CD4:CD8 T cell ratio and this dysregulation positively correlates with maternal viral load (15).

As labor is an inflammatory process, it has been proposed that in addition to resident placental immune cells, during late pregnancy, there is an increased influx of maternal leukocytes recruited to reproductive tissues including the cervix, myometrium and maternal decidua (16, 17). This then results in a pro-inflammatory state which leads to labor and delivery. It is therefore thought that the premature activation of these pro-inflammatory signals results in premature labor arising from a breakdown in maternal-fetal tolerance which can subsequently result in PTB (11). Research on placental immune dysfunction in pregnant women with HIV is limited. In addition, studies on the placental origin of PTB in women with HIV are lacking. However, given the increased rates of PTB in women with HIV, it is important that the perturbed pathways within the placental microenvironment are elucidated. Here, we aim to review evidence from studies that point toward the placental origin of spontaneous PTB and discuss possible ways maternal HIV infection could increase this risk. We focus on key cellular players in the maternal decidua including natural killer cells, CD4+ T cells including CD4+ regulatory T cells, CD8+ T cells as well as macrophages.

## Risk of Preterm Birth Among Women With HIV

Well-established risk factors for spontaneous preterm birth (PTB) include previous PTB, uterine anomalies, twin pregnancies and genetic factors (18–21). Infections can also increase this risk as discussed herein; maternal HIV infection and the use of antiretroviral therapy (ART) have been associated with an increased risk of PTB.

A meta-analysis of 52 cohort studies including 24 studies from Africa, 11 from USA, 2 from Europe and 6 from Asia found that maternal HIV infection was significantly associated with PTB (pooled odds ratio [OR]:1.56, 95% confidence interval [CI]:1.49–1.63). In this study, the prevalence of infants born preterm ranged from 5.2 to 73.0% in women with HIV and, 2.2 to 32% in women with no HIV, although no distinction was made between spontaneous and caregiver initiated PTB (5). The authors reported that ART usage did not significantly change the associations of maternal HIV exposure with PTB (5). One key limitation here however was that there was insufficient information from the individual studies to support the analysis of the data stratification based on specific ART regimens, time of ART initiation and duration on treatment (5).

A meta-analysis of 14 prospective and 8 retrospective cohort studies from 11 countries in sub-Saharan Africa, one country in Asia, one in Europe and the other in the Americas found that maternal HIV infection was associated with an increased risk of PTB; prospective studies (risk ratio [RR]:1.5, 95% CI: 1.24–1.82) and retrospective studies (RR: 1.82, 95% CI: 1.41–2.34) (6). The same authors also performed a meta-analysis of 12 retrospective cohort studies which demonstrated that maternal HIV infection increased the risk of preterm low birthweight (RR: 3.25, 95% CI: 2.12–4.99). However, there was no distinction made between spontaneous and caregiver initiated PTB, furthermore the data was not stratified by specific ART regimen (6). In addition, a retrospective analysis of data from a cohort study in Kenya on pregnant women with HIV receiving a short-course of Zidovudine for the prevention of vertical transmission found that elevated maternal plasma (OR: 2.1, 95% CI: 1.1–3.8), cervical HIV-1 RNA levels (OR: 1.6, 95% CI: 1.1–2.4) and maternal CD4 of <15% (OR: 2.4, 95% CI: 1.0–5.6) were most associated with an increased risk of spontaneous singleton PTB (22).

Certain ART regimens have also been associated with an increased risk of PTB [reviewed in detail by Short et al. (23)]. A meta-analysis of 10 studies showed that exposure to protease inhibitor (PI) based ART during pregnancy was associated with an increased risk of PTB (pooled OR: 1.32, 95% CI: 1.04–1.59) although no distinction was made between spontaneous and caregiver initiated PTB (24). The Promoting Maternal and Infant Survival Everywhere (PROMISE) trial showed that Zidovudine (ZDV)-based ART was associated with significantly higher rates of PTB compared to ZDV monotherapy (20.5 vs. 13.1%) and, combination ART comprising of Tenofovir Disoproxil Fumarate (TDF) was associated with significantly higher rates of very PTB compared to ZDV-based ART (6 vs. 2.6%, *p* < 0.04) again, no distinction was made between spontaneous and caregiver initiated PTB (25). More recently, a network meta-analysis on 35 studies including eight randomized control trials, 1 case control and 26 cohort studies showed that PTB was increased in women taking ZDV administered with Lamivudine and Indinavir compared to ART naïve women (OR: 69.59, 95% CI: 2.49–4,982) although here, the authors reported significant heterogeneity between the studies included in the analyses, no distinction was made between spontaneous and caregiver initiated PTB (26).

Timing of ART initiation has also been associated with an increased risk of PTB. A systematic review and meta-analysis on 11 studies by Uthman et al. (27) reported that women who started ART before conception were more likely to deliver preterm (pooled RR 1.20, 95% CI: 1.01–1.44) or very preterm (pooled RR 1.53, 95% CI: 1.22–1.92) than those who began ART after conception although no distinction was made between spontaneous and caregiver initiated PTB. The mechanisms underlying the increased risk of PTB in women who initiate ART before pregnancy are not well-understood. However, in a recent study on pregnant women initiating ART either before or during pregnancy, we found that women who initiated ART before pregnancy were more likely to have maternal vascular malperfusion (MVM) (13). Furthermore, MVM was significantly increased in placentae from women who delivered preterm (13). MVM refers to a constellation of gross and histologic findings that represent injury affecting maternal vasculature and circulation in the placenta and is linked to adverse birth outcomes including but not limited to PTB (28). It is important to note however, that further research is needed in this area because there is some debate on whether the effect of timing of ART initiation on birth outcomes is a true effect or due to selection bias (29–31).

Taken together, the data above suggests that in utero maternal HIV and/or ART are likely associated with increased risk of PTB, further justifying the need for a better understanding of underlying mechanisms.

## HIV Infection At the Maternal Fetal Interface

HIV can cross the placenta and the virions can either be cell-associated, antibody-associated or free (32, 33). The virions can interact directly with the tissue resident immune cells including Hofbauer cells and decidual macrophages which express the HIV receptor CD4 and co-receptors C-C chemokine receptor 5 (CCR5), C-X-C chemokine receptor 4 (CXCR4) and Dendritic Cell-Specific Intercellular adhesion molecule-3-Grabbing Non-Integrin (DC-SIGN) (32, 33). In fact, there is evidence that viral replication may occur locally within the placenta based on phylogenetic studies demonstrating genetic divergence between placental and systemic maternal HIV quasi species (34, 35). In this section we review evidence that from studies that demonstrate how the placenta “naturally” protects the fetus from viral infection *in utero* (33, 36, 37). This protection is mediated by key placental immunocytes including decidual NK (dNK) and Hofbauer cells which inhibit HIV infection at the maternal fetal interface.

Decidual NK (dNK) cells from early gestation have been shown to inhibit infection of decidual macrophages, highlighting the role of robust innate responses in the control of HIV transmission (36). Others have shown that decidual culture supernatants contain the CC chemokine ligands 3 and 4 (CCL3 and CCL4) which inhibit HIV infection by binding to the HIV co-receptor CCR5, thereby decreasing the risk of infection (38). Hofbauer cells have also been shown to constitutively express elevated concentrations of regulatory cytokines which inhibit HIV replication *in vitro* and these cells also possess intrinsic antiviral properties [reviewed in detail by Johnson and Chakraborty (39)]. This “placental protection” against infection is not unique to HIV and has been demonstrated for other viral pathogens including human cytomegalovirus (HCMV), hepatitis C virus (HCV) and herpes simplex virus (HSV) (40–42).

The risk of vertical transmission seems to increase with an increase in gestational age; with lowest and highest transmission in the first trimester and third trimester respectively (33, 43, 44). A recent report by Johnson et al. (33) provides mechanistic evidence in support of this observation. In their study, early gestation Hofbauer cells (HCs) displayed a more activated phenotype compared to term cell isolates. Interestingly, early gestation HCs expressed higher levels of CCR5 compared to term, but the term HCs were more susceptible to HIV replication. The early/mid gestation HCs had increased secretion of anti-inflammatory cytokines, chemokines and more robust antiviral responses.

In addition to these robust immune responses in early pregnancy, the uptake of maternal antiretroviral therapy (ART) has been shown to significantly reduce the risk of vertical transmission, even if taken in the third trimester (45). However, even in the absence of vertical transmission, maternal HIV infection is associated with an altered immune landscape in the decidua and villous tissue (15). In addition, placentae from women with HIV have been shown to have increased features of placental insufficiency including maternal vascular malperfusion, and features of acute and chronic inflammation (12). Furthermore, HIV exposed uninfected (HEU) neonates have an increased risk of morbidity and mortality, an increased risk of being born preterm and suboptimal behavioral and neurological development compared to their HIV unexposed uninfected (HUU) counterparts (46, 47). HEU also have an altered immune landscape characterized by lower CD4+ T cell counts at birth and increased proportions of differentiated immune cells suggestive of antigen experience *in utero* (48, 49).

Therefore, despite all efforts by the placenta to “fight” the viral infection and maternal ART to reduce the risk of vertical transmission, maternal HIV infection is still associated with perturbances at the maternal fetal interface and, with maternal and neonatal implications. This may underlie the increased rates of PTB seen in women with HIV. In the next section we focus on key cellular players in the decidua that may be altered in pregnant women with HIV, thereby increasing the risk of PTB.

## Decidual Immune Cells, Preterm Birth and HIV

**Figure 2:**
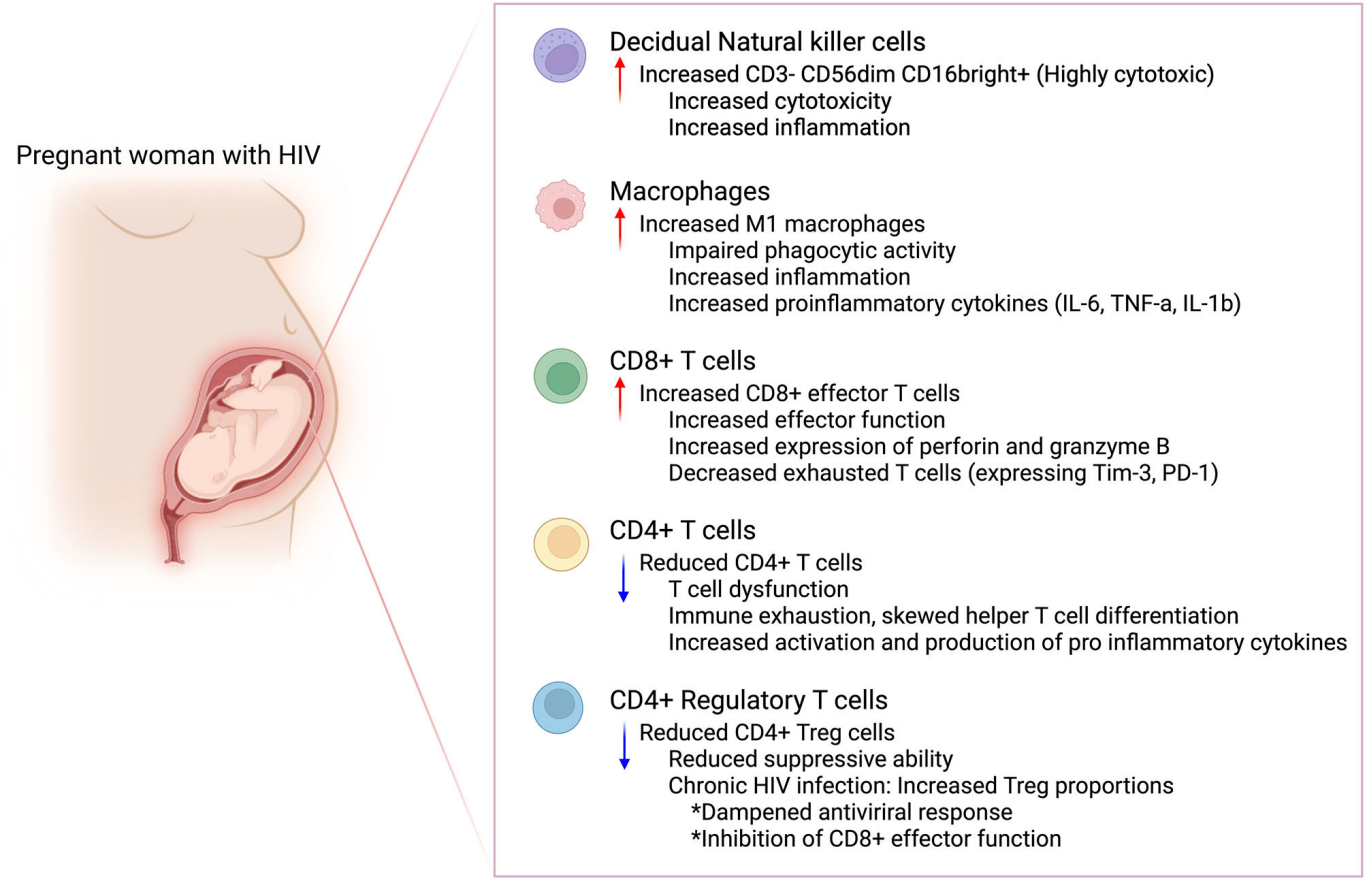
Possible immune perturbances in the maternal decidua in women with HIV. Maternal HIV infection could lead to immune perturbances in the maternal decidua. This immune dysfunction could increase the risk for preterm birth in women with HIV. Created in Biorender.com.

Human pregnancy requires effective crosstalk between two genetically dissimilar individuals in order to maintain and support the developing fetus (50). This requires a complex balance where the maternal immune system has to tolerate the fetus while simultaneously providing the requisite protection against potential infectious threats (51). The success of these immune interactions is based on the unique cellular immune repertoire within the placenta which is comprised of different immune cell subsets that play an important role during pregnancy. These roles include inter alia, maintaining maternal fetal tolerance, providing support for placental and fetal development as well as participating in the inflammatory processes that accompany labor (52). Perturbances in these immune cell interactions, subset proportions or functions has been associated with adverse birth outcomes including the premature induction of labor which can lead to preterm birth (PTB). This is discussed below and summarized in Figure 2.

### Decidual Natural Killer Cells

Natural killer (NK) cells are innate lymphoid cells that were named “natural” killer cells because of their ability to destroy tumor cells without prior sensitization (53). In humans, NK cells are present in peripheral blood (pbNK), but are also widely distributed across a number of tissues, “tissue resident” NK (trNK) cells (54). In this regard, decidual NK (dNK) cells are a unique tissue resident subset found in decidual tissue. Peripheral blood NK cells comprise approximately 10% of total leukocytes and are broadly classified into either CD3^−^ CD56^dim^ CD16^bright^ cells which are highly cytotoxic or CD3^−^ CD56^bright^ CD16^dim−^ cells which show weak cytotoxicity (55). Majority (~90%) of the pbNK are CD3^−^ CD56^dim^ CD16^bright^. On the other hand, dNK cells are predominantly CD56^bright^ CD16^dim−^, and therefore portray minimal cytotoxicity (56).

Within the larger dNK pool, Vento-Tormo et al. (57) described three dNK subsets based on their different phenotypic and functional characteristics, dNK1, dNK2 and dNK3. These cells are present in the decidua throughout the course of gestation however, their proportions are highest during the first trimester (~70% of total lymphocytes) (56). During this phase of pregnancy, dNK regulate trophoblast invasion by producing chemokines including interleukin 8 (IL-8) and interferon gamma-induced protein 10 (IP-10) which bind to the chemokine receptors, CXCR1 and CXCR3 expressed by invasive extravillous trophoblasts (EVTs) (58). These dNK cells also express vascular endothelial growth factor (VEGF-C), Arginase 1 and 2 and transforming growth factor beta (TGF-β) which are important for EVT invasion and subsequent spiral artery remodeling (59, 60).

Decidual NK cell numbers diminish as pregnancy progresses however compared to first trimester NK, term dNK are phenotypically distinct when compared to first trimester subsets. Term dNK become less granular (61, 62). The change in dNK granularity over the course of gestation suggests a change in the functional role of dNK cells. In addition, term dNK have a distinct cytotoxic profile and have been shown to upregulate a series of genes including *Interferon gamma (IFN-*γ*), CD69, integrin subunit beta 2* (*ITGB2*) and *NKp80* which are involved in the induction and modulation of an array of immune responses including immune activation (63).

The specific function of term dNK remains to be determined and to the best of our knowledge, there have been no studies linking dNK phenotype or function to HIV-associated preterm labor/birth. However, they may play a role in labor as Sindram-Trujillo and colleagues reported a significantly higher proportion of CD56^dim^ CD16^+^ NK cells in term placentae from women with spontaneous vaginal delivery when compared to women who delivered following elective cesarean section (64). Pique-Regi et al. also observed that chorioamnionitic membranes, basal plate and villi contained NK cells at term and further reported that placentae from women with spontaneous preterm labor had upregulated expression of single-cell signatures of NK cells when compared to gestational-age matched control placentae from women without labor (65).

Furthermore, and in line with HIV-associated preterm labor/birth, there is evidence that suggests dNKs become highly cytotoxic subset upon encountering a pathogen (36, 66, 67). This shift may lead to a break in maternal fetal tolerance thus increasing the risk of preterm birth in women with HIV. Quillay et al. (36) recently reported that dNK efficiently inhibit HIV infection of decidual macrophages *in vitro*. Here, the authors demonstrate that cellular contacts between decidual macrophages and dNK are necessary for optimal control of infection and they further provide evidence for the role of IFN-γ in the control of infection. This is quite interesting because dNK are classically known to be weakly cytotoxic, but these findings suggest that upon encountering a pathogen there is a notable functional shift. This is not a feature unique to HIV infection as dNK cells exposed to human cytomegalovirus (HCMV) infected cells have been shown to display phenotypic changes and acquire cytotoxic function (66). Decidual NK cells are able to kill fibroblasts infected by human cytomegalovirus (HCMV) *in vitro* suggesting that dNK control *in utero* transmission of HCMV (66). *Toxoplasma gondii* infection is also associated with a significant increase in cytotoxic dNK cell numbers *in vitro* (67). This may also explain why the rates of vertical transmission for viral, bacterial and parasitic infections are quite low in the first trimester as this immune protection coincides with a significant increase in dNK at the maternal fetal interface (68, 69).

It would be worthwhile to explore the phenotypic and functional alterations in dNK from women with HIV, particularly in placentae from women with PTB. As seen with HCMV, HIV exposure may result in phenotypic and functional shifts of dNK from being weakly cytotoxic (CD3^−^ CD56^bright^ CD16^dim−^ cells) to a highly cytotoxic subset. This may result in poor placental development including defective deep transformation of the myometrial segments of the spiral arteries. Defective deep placentation can result in placental features like maternal vascular malperfusion which is associated with an increased risk of PTB (13, 28). Indeed, studies have shown that women with larger populations of cytotoxic NK populations in the uterine mucosa may be at an increased risk of infertility and related disorders owing to increased inflammation (70). It may also be that HIV infection alters the proportion of NK cell populations in the decidua. Studies have demonstrated that HIV can impact trNK dynamics from other mucosal sites including the gut (71).

### Macrophages

Macrophages are innate myeloid immune cells which strategically reside in tissues where they detect, process and present antigens (72). Macrophages can be classified into either M1 or M2 based on their phenotypic and functional properties. M1 macrophages are classically activated, inflammatory cells induced by IFNγ and lipopolysaccharide (LPS) (73). They express the transcription factor interferon regulatory factor 5 (IRF5), the co-stimulatory molecules CD80, CD86 and the FCγ receptor 1, CD64 (74). M1 macrophages secrete pro-inflammatory cytokines including IL-12, IL-23 as well as nitric oxide (NO) and reactive oxygen species (ROS) (75). M2 macrophages on the other hand express the scavenger receptor CD163 and the mannose receptor and are a regenerative subset induced by Th2 cytokines including IL-4, IL-13, IL-10 and are involved in immune tolerance and tissue remodeling (76).

Decidual macrophages are formed from peripheral blood monocytes that migrate into the tissue in response to pro-inflammatory, metabolic and immune stimuli (73). During the peri-implantation period, there is a predominance of an M1 immune profile. Soon after implantation, the immune profile shifts toward a mixed M1/M2 profile to support trophoblast invasion. These “*mixed*” cells have been shown to simultaneously express M1 (CD64, CD80, CD86) and M2 (CD126, CD206) markers (77). During this stage, the macrophage proteolytic enzymes, matrix metalloproteinases (MMP)-7 and MMP-9 are involved in preparing the spiral arteries for trophoblast mediated remodeling by disrupting and loosening the vascular smooth muscle cell layers (78).

On completion of placental development, the macrophages are then polarized to M2 providing a tolerogenic immune profile required for optimal fetal development (79). Evidence to support the importance of macrophages in the maintenance of tolerance was determined in a functional study where the authors demonstrated decidual macrophages suppress T cell IFNγ production (80). Furthermore, histological analysis showed an increased proportion of macrophages surround apoptotic cells suggesting that decidual macrophages can induce apoptosis of damaged cells which could otherwise be lethal to the developing fetus (81).

To the best of our knowledge, there have been no studies linking macrophage phenotype or function to HIV-associated preterm labor/birth. However, aberrant macrophage function and numbers have been associated with adverse birth outcomes. M1 macrophages which are classically inflammatory have been shown to be decreased in decidua from normal pregnancies but not in cases of spontaneous abortions or unexplained recurrent pregnancy loss (82, 83). Furthermore, the co-culture of macrophages with regulatory T cells results in increased production of IL-10, which has been shown to be decreased in decidua from women with unexplained recurrent pregnancy loss (84). This demonstrates the role of macrophages in mediating the generation of a tolerogenic microenvironment to support fetal development.

Macrophages are thought to play a role in the labor process, and it has been shown that toward the end of pregnancy, the M2 immune profile shifts toward a pro-inflammatory, counter-regulatory landscape (11). The pro-inflammatory macrophages produce increased levels of IL-1β, IL-6, TNFα, and ROS all of which have been associated with the induction of labor (85, 86). These pro-inflammatory cytokines can regulate the release of MMPs by fetal membranes which has been shown to be one of the mechanisms by which macrophages contribute to the rupture of membranes. MMP-9 levels are significantly increased in the fetal membranes during preterm labor, labor and preterm premature rupture of membranes (PPROM) (87, 88). Macrophage tissue density has also been shown to be lower in women who delivered at term without labor compared with women who delivered term and preterm with labor although in this study, the macrophages were defined using CD68+, a pan-macrophage marker (89).

It remains to be determined how macrophage phenotype or function could possibly increase the risk of preterm labor/birth in women with HIV but decidual macrophages have been investigated in HIV infection because these cells are the main HIV R5-tropic target cells in the decidua (90). Costa and colleagues (91) demonstrated that IFN-γ in the decidual tissue restricts HIV-1 infection of decidual macrophages by mechanisms involving cyclin-dependent kinase inhibitors. In addition, the activation of Toll-like receptors 7 and 8 expressed by the decidual macrophages restricted viral replication. Quillay et al. demonstrated that dNK cells inhibit decidual macrophage infection through soluble factors including IFN-γ and through cellular contact between the two cell populations (36). Furthermore, decidual macrophages have been shown to express the cellular restriction factor sterile alpha motif domain HD domain-containing protein (SAMHD1) which is known to restrict HIV infection in myeloid cells and in resting CD4+ T lymphocytes (77).

Further research is warranted in determining how decidual macrophages are linked to the risk of preterm labor in women with HIV. Tissue resident macrophages from other sites are known to be persistently infected, therefore even though ART generally reduces the burden of HIV disease, the low-level residual inflammation resulting from persistent infection may contribute to progressive disease and chronic inflammation (92). In addition, these macrophages may have impaired phagocytic activity as has been demonstrated with alveolar macrophages in both ART-treated and -untreated individuals (93). Furthermore, it would be of interest to explore whether a highly inflammatory microenvironment in women with HIV may result in the polarization of macrophages toward a more inflammatory M1 phenotype as observed. Lastly, inflammation is associated with an increased influx of monocytes to the site of tissue damage. Placentae from women with HIV present with increased features of acute and chronic inflammation, therefore it is worth exploring whether maternal HIV infection results in an increased influx of monocytes to the decidua, whether the local environment polarizes the monocytes to M1 and how this would possibly lead to preterm labor/birth.

### CD8+ Effector T Cells

CD8+ T cells are a heterogeneous cytotoxic lymphocyte subset that play a major role in fighting infections particularly viruses and intracellular pathogens (94). These cells are highly migratory therefore, their phenotypic and functional characteristics have been shown to be tissue specific (95). In this regard, decidual CD8+ T cells have distinct phenotypic and functional features compared to peripheral blood CD8+ T cells (96). Approximately 50% of peripheral blood CD8+ T cells are naïve, however, a large majority of decidual CD8+ T cells are effector T cells which indicate they are antigen experienced (97). Peripheral blood CD8+ T cells express high levels of cytolytic molecules including perforin and granzyme B but the decidual subsets express significantly lower levels of these cytolytic molecules (98). This is despite the fact that mRNA analysis demonstrated that perforin and granzyme B expression is higher in decidual CD8+ T cells compared to peripheral blood CD8+ T cells, suggesting that post-transcriptional modifications ensure these cells have reduced cytotoxicity against fetal antigens (99, 100).

Decidual CD8+ T cells can directly recognize allogeneic MHC class I molecules and are therefore considered a direct threat to fetal survival. However, there are several mechanisms in place to ensure these cells do not disrupt fetal development. One mechanism is the reduced expression of cytolytic molecules mentioned above. In addition, fetal tissues, specifically the invading extravillous trophoblast cells (EVT) lack the expression of the classical HLA-A and HLA-B molecules which means that the CD8+ T cells are less likely to attack EVTs compared to an organ transplant (96). Rather they express HLA-G, a non-classical HLA molecule which interacts with leukocyte Ig-like receptor family B (LILRB) receptors that exhibit immunosuppressive behavior upon their activation (101, 102). Furthermore, decidual CD8+ T cells display exhausted and senescent phenotypes which means they are less likely to exert their cytolytic activity and disrupt normal gestation [reviewed in detail by Millar et al. (103)].

The downregulation of the effector T cell function is important for the maintenance of maternal fetal tolerance. However, a number of studies have suggested that labor is associated with the restoration of CD8+ T cell effector function. Decidual CD8+ T cells isolated from women who underwent the physiological process of labor at term were found to have increased expression of perforin and granzyme B when compared to women who were not in labor (104). Women who delivered preterm with labor were also shown to have increased decidual CD8+ expression of perforin and granzyme B compared to those who delivered preterm with no labor (104). These findings are supported by *in vitro* studies and single-cell analyses suggesting that the physiological process of labor is associated with the amplification of effector function (65, 97). Mechanistic studies have also demonstrated that blockade of Tim-3 and PD-1, markers of T cell exhaustion results in fetal loss (105). Moreover, Slutsky et al. (106) demonstrated that placentae with features of acute inflammation from women who delivered preterm had reduced proportions of exhausted CD8+ T cells. These findings suggest that inflammation can reactivate exhausted T cells, and this could result in adverse outcomes including preterm labor.

The dynamics of CD8+ T cells in HIV infection have been studied in the peripheral blood and decidua. It is now well-established that acute HIV infection is associated with expansion of the effector systemic CD8+ T cell pool and although these cell numbers decline slightly following the initiation of antiretroviral therapy (ART), they remain elevated in most individuals even after years of treatment (107, 108). A similar occurrence is noted in the decidua, as it has been recently shown that decidual CD8+ T cells are increased in placentae from women with HIV at term and these proportions correlate with maternal viral load pre-ART (15). Women with high viral loads prior to ART initiation are more likely to have increased CD8+ T cells in the maternal decidua at delivery.

Interestingly, the marked activation of the CD8+ T cell pool in individuals with HIV is only attributed in part to HIV-specific CD8 T cells (108). Less than 10% of the activated CD8+ T cells are HIV-specific in acute and chronic infection owing to the fact that CD8+ T cells reactive to other pathogens are expanded in individuals with HIV infection; some of which may be due to reactivation of latent pathogens including cytomegalovirus (CMV) and Epstein-Barr virus (EBV) (109–111). CD8+ T cells from people with HIV are largely effector memory cells compared to the predominant naïve and central memory CD8 pool in healthy individuals (112). Furthermore, these CD8+ T cells exhibit features of T cell exhaustion and immunosenescence and are functionally impaired as effector T cells with limited proliferative capacity in response to antigen and reduced cytokine production and ART only partially restores this functionality.

It remains to be determined how decidual CD8+ T cell dynamics are linked to HIV-associated preterm birth. As mentioned above, inflammation can reactivate CD8+ T cell effector function during pregnancy and this can result in preterm labor/birth. Chronic inflammation is a hallmark feature of HIV infection due to persistent viral replication, reactivation of HIV reservoirs, recurrence of latent infections and loss of gut integrity (113). Furthermore, HIV is associated with a systemic increase in pro-inflammatory cytokines including IL-6, IL-1β and TNF-α. These are all factors that may significantly increase the risk of preterm labor/birth. In addition, as it has been shown that only a small percentage of the expanded CD8+ T cell pool in the peripheral blood is HIV-specific, it would be worth determining the antigen specificity of the CD8+ T cells in the decidua in HIV-exposed placentae. Furthermore, the increase of systemic CD8+ T cells in HIV infection may be associated with an increased influx of CD8+ T cells into the decidua. This may increase the risk of trophoblast cytotoxicity and pregnancy complications. The role of CD8+ T cells as central mediators in the provision of protective immunity to the fetus can also not be ignored and it is therefore important that further research on the impact of impaired CD8+ T cell function in women with HIV is conducted.

### CD4+ T Cells

CD4+ T cells are key players of the adaptive immune response as they play an integral role in the development and activation of B cells and CD8+ T cells. CD4+ T cells are a heterogeneous subset and include T helper 1 (Th1), Th2, Th17, Th22, regulatory T cells (Tregs) and T follicular cells (Tfh), reviewed in detail by Chatzileontiadou et al. (114). In the context of pregnancy, Th1 and Th2 have been extensively studied resulting in the well-known classic model of immune regulation that proposes a shift from a Th1 to a Th2 immune response for the maintenance of maternal fetal tolerance to support fetal development (115).

Th1 cells are involved in the pathogenicity of organ-specific autoimmune diseases and express the cytokines interferon gamma (IFN γ), tumor necrosis factor alpha (TNFα) and interleukin 2 (IL-2) which promote inflammation (116). These cells are therefore thought to play a role in pathological pregnancy and likely drive premature expulsion of the fetus particularly because they are the primary CD4+ T cell subset that drive surgical allograft rejection (117, 118). However, recent evidence shows that not all Th1 responses are abated during pregnancy. In fact, a controlled Th1 immune response is beneficial during the peri-implantation period (119, 120). TNFα, IFN γ and IL-2 have a regulatory role involving vascular remodeling and the inhibition of excessive trophoblast invasion (119–122). A delicate balance between pro- and anti-inflammation is however required because these same cytokines have been associated with the immunopathology of various obstetrics complications including preeclampsia (123).

Soon after implantation, there is a shift toward a Th2 dominant immune response (115). Th2 cells secrete anti-inflammatory cytokines including IL-4, IL-10 and IL-13 which repress the development of the pro-inflammatory immune responses (Th1 and Th17) and promote maternal fetal tolerance (115). Women with recurrent pregnancy losses and miscarriages have been shown to have a Th1 bias compared to Th2 (124, 125). Similar adverse outcomes have been reported in mice where the induction of Th1 cytokines results in abortion (126). Additionally, administration of IL-10, a Th2 anti-inflammatory cytokine has been shown to prevent pregnancy loss (127). A Th2-skewing of T cell responses is therefore considered favorable during pregnancy until later in pregnancy when an inflammatory and less regulatory immune response is required for the induction of labor (115).

The involvement of T cells during labor has been investigated. CD4+ T cells have been shown to be more abundant in term than in preterm gestations without labor (128). The recruitment of CD4+ T cells to the rupture zone of the fetal membranes via chemotaxis facilitated by CXCL10 and CCL5 has been shown during term labor (128–130). Furthermore, decidual CD4+ T cells express activation markers including CD25 and secrete pro-inflammatory cytokines including TNFα, IL-1 and matrix metalloproteinase 9 (MMP-9) during spontaneous labor at term suggesting that a pro-inflammatory response may be associated with parturition (128, 131).

CD4+ T cells have been well-characterized in HIV infection and in fact one of the hallmark features of HIV infection is the gradual loss of CD4+ T cells and the imbalance of CD4+ T cell homeostasis due to the fact that HIV preferentially infects and destroys CD4+ T cells (132). It has also been recently shown that HIV infection is associated with a dysregulated T cell landscape in the maternal decidua; women with HIV have lower proportions of CD4+ T cells compared to women with no HIV (15). The depletion of CD4+ T cells in HIV infection is mainly thought to be due to direct HIV infection. However, this loss may also be attributed to chronic immune inflammation secondary to chronic exposure to microbes translocated across epithelial barriers including the gut and fibrotic change to lymphoid tissue during primary HIV infection (133, 134). Virus-specific CD4 T cell dysfunction has also been studied and this has been shown to result from a combination of immune exhaustion and skewing in helper T cell lineage differentiation (135). Antiretroviral therapy restores CD4+ T cell numbers however, in a setting of chronic immune activation and immune dysregulation, CD4+ T cell dysfunction is not fully restored resulting in the eventual failure of CD4+ T cell homeostasis, decline in critical effector populations and a subsequent increase in the risk of acquiring opportunistic infections (8).

Women with HIV may be at an increased risk of PTB owing to these perturbances. As discussed above HIV infection is associated with a chronic state of immune activation which is characterized by a high T cell turnover and elevated proinflammatory cytokines and chemokines including type 1 interferons, IL-1α, IL-1β, IL-6, TNFα all of which have been reported to be increased in spontaneous term and preterm labor. Furthermore, the persistent immune activation in HIV infected individuals prevents the establishment of memory CD4+ T cells which are required, not only for HIV-specific immunity, but also for the maintenance of maternal fetal tolerance. These features of a perturbed immune footprint in women with HIV may contribute a break in maternal fetal tolerance resulting in the induction preterm labor.

### CD4+ Regulatory T Cells

CD4+ Regulatory T cells (Tregs) are a distinct T cell subset classically defined as CD4^+^ CD25^bright^ CD127^lo^ FoxP3^+^. Tregs are known to be potently immunosuppressive and have therefore been studied in pregnancy as they play a critical role in the establishment and maintenance of maternal fetal tolerance (136, 137). It is well-established that both peripheral and decidual Treg numbers significantly increase during pregnancy and defects in maternal Treg frequency and function have been associated with pregnancy complications including recurrent pregnancy loss, preeclampsia and preterm birth (138–141). However, the role of Tregs in HIV-associated preterm labor/birth is yet to be determined. Previous studies have largely focused on the role of Tregs in the establishment and maintenance of maternal fetal tolerance during the course of gestation but there is now an increasing interest on the role Tregs play toward the end of gestation, particularly during labor. These initial studies provide a foundation for further studies which can contribute toward the elucidation of how altered Treg numbers or function may be linked to an increased risk of preterm birth in women with HIV.

An in-depth investigation of decidual FoxP3^+^ Tregs in women undergoing cesarean sections without any signs of labor, in early labor or in advanced labor confirmed that Tregs are present at the maternal fetal interface at term (142). Interestingly, the authors reported that the proportions of the decidual Tregs declined as labor progressed, suggesting that labor is associated with alterations in Tregs at the maternal fetal interface (142). Salvany-Celades et al. (143) defined three distinct Treg subsets in the decidua based on the expression of CD25, PD1 and TIGIT in first trimester and third trimester specimens. The authors demonstrated that the suppressive ability of decidual Tregs was significantly higher in first trimester decidual Tregs compared to Tregs in term decidua. Furthermore, a recent report demonstrated decidual Tregs exhibit suppressive activity, however in this study, functional Tregs were reduced at the maternal fetal interface in a subset of women with idiopathic preterm labor/birth compared to women who underwent the physiological labor process at term (141). Here, the authors further demonstrated that depletion of functional Tregs in mice is associated with PTB and adverse neonatal outcomes, which can be rescued by the adoptive transfer of Tregs. Worth noting is that the depletion of Tregs did not induce PTB in all the dams however, the risk of having a PTB was significantly increased upon encountering an inflammatory agent.

Zeroing in on the effect of HIV, both decidual and systemic Tregs from pregnant women with HIV remain under investigated. One study found that PWH had increased proportions of circulating Tregs in early pregnancy compared to pregnant women not living with HIV (PWNH) but an inverse during late pregnancy (144). In a second study, Tregs in PWNH peaked during the second trimester of pregnancy but this was not observed in PWH (145). These findings suggest that HIV may alter the dynamics of circulating Tregs during pregnancy although the data available is limited. This therefore leads us to infer from Treg dynamics in non-pregnant individuals which have been extensively studied and reviewed (146–148).

The evidence available shows that Treg frequency is associated with maternal viral load and HIV disease progression (149). Absolute Treg numbers are decreased in the circulation in both acute and chronically infected individuals however, the percentage of Tregs in chronic infection is relatively increased (150, 151). The percentage increase in chronically infected individuals may be attributed to an increase in proliferation or it may be that these cells are relatively resistant to HIV-associated cell death (152). The increase in percentage of Tregs means that unlike CD4+ T cells which rapidly decline in HIV infection, Tregs appear to be relatively spared. However, it is not known whether HIV affects Treg suppressive function during pregnancy and if the decline in absolute Treg numbers as seen in acute and chronically infected individuals could lead to preterm labor/birth.

In terms of modulation of HIV infection, Tregs can either be beneficial or detrimental. In acute infection, Tregs have been shown to inhibit T cell activation before HIV-specific immune responses are fully activated and thereby reducing the number of target cells for HIV infection (153). However, in chronic infection, the increase in Tregs may dampen the antiviral immune response leading to decreased clearance of HIV and thus persistence of infection. Indeed, Tregs have been shown to inhibit CD8+ effector T cell proliferation and differentiation (154). In addition, individuals with chronic HIV infection have been shown to have Treg subsets expressing high levels of immune-modulatory molecules that can broadly inhibit T cell responses (154, 155). Furthermore, Tregs are potentially susceptible to both HIV R5-tropic and X4-tropic infection as they express the chemokine receptor 4 (CXCR4) and chemokine receptor 5 (CCR5) although conflicting findings have been reported on the effect HIV-infection has on Treg function and phenotype (156). Some studies show that infected Tregs are not functionally altered when infected while others have reported infected Tregs have reduced immunosuppressive potency (157–159). To support the latter theory, studies have shown that Tregs infected with HIV X4-tropic strains have decreased FoxP3 expression and thereby less suppressive (160). In addition, Tregs from people with HIV with high viral loads were shown to have reduced expression of CD25 (IL-2Rα) (161).

A better understanding of the dynamics of Tregs in women with HIV is needed particularly because alterations in Treg numbers and function have been associated with adverse birth outcomes including PTB. In addition, as Tregs are increased in chronic infection, this may likely result in dampening of the anti-viral response which could predispose the infant to an increased risk of acquiring not only HIV but other opportunistic infections including the classic group of perinatal infections, TORCH: toxoplasmosis, other (syphilis), rubella, cytomegalovirus and herpes simplex which may have adverse consequences for the fetus (162).

## Concluding Remarks

The role of decidual cells in HIV-associated preterm labor/birth remains to be determined however, this review demonstrates that, there is an increasing interest on the role of these cells in the labor process and HIV. Decidual NK cells have been shown to display phenotypic and functional shifts in women who have spontaneous vaginal deliveries and dNK cells also become highly cytotoxic in the presence of a number of pathogens including HIV. Decidual macrophages on the other hand are also of interest as they are the main HIV R5-tropic target cells in the decidua, but it remains to be determined how HIV impacts the macrophage polarization and function and how this would impact the risk of preterm birth. CD8+ T cells have been shown to be increased in placenta from women with HIV but their functional alterations in a HIV/ART exposed placental microenvironment is yet to be established. CD4+ T cells in placentae from women with HIV are significantly decreased but again like with the CD8+ T cells their functional alterations in a HIV/ART exposed placental microenvironment is yet to be established.

Chronic inflammation is a hallmark feature of HIV infection; inflammation can reactivate effector T cell function during pregnancy, and this can result in preterm labor/birth. The persistent immune activation in HIV infected individuals could also possibly prevent the establishment of memory T cells which are required, not only for HIV-specific immunity, but also for the maintenance of maternal fetal tolerance. Taken together, it is likely that features of a perturbed immune footprint in the placentae of women with HIV may underlie the increased risk preterm labor/birth associated with maternal HIV/ART. It is therefore important that additional efforts are geared toward a better understanding of the dynamics of decidual cells in women with HIV particularly in the context of preterm labor/birth.

## Author Contributions

NI wrote the review. MM modified the review. Both authors contributed to the article and approved the submitted version.

## Funding

NI is a research associate supported by the National Institute for Health Research (NIHR) (17/63/26) using UK Government aid to support global health research.

## Author Disclaimer

The views expressed in this publication are those of the authors and not necessarily those of the NIHR or the UK Department of Health and Social Care.

## Conflict of Interest

The authors declare that the research was conducted in the absence of any commercial or financial relationships that could be construed as a potential conflict of interest.

## Publisher's Note

All claims expressed in this article are solely those of the authors and do not necessarily represent those of their affiliated organizations, or those of the publisher, the editors and the reviewers. Any product that may be evaluated in this article, or claim that may be made by its manufacturer, is not guaranteed or endorsed by the publisher.
